# Supplementary material for: Streptococcus pyogenes Infection and the Human Proteome with a Special Focus on the Immunoglobulin G-cleaving Enzyme IdeS
Source: Mol Cell Proteomics. 2018 Mar 6;17(6):1097–111. doi: 10.1074/mcp.RA117.000525 (PMC5986240; doi:10.1074/mcp.RA117.000525)
Supplement: Supplemental Data [file supp_17_6_1097__index.html]

Streptococcus pyogenes infection and the human proteome with a special focus on the IgG-cleaving enzyme IdeS. — Streptococcus pyogenes and the human proteome — Streptococcus pyogenes Infection and the Human Proteome with a Special Focus on the Immunoglobulin G-cleaving Enzyme IdeS — Streptococcus pyogenes and the Human Proteome — Supplemental Data 

# *Streptococcus pyogenes* Infection and the Human Proteome with a Special Focus on the Immunoglobulin G-cleaving Enzyme IdeS

## Supplemental Data

- Suppl.Tab.S1-S5 - Supplemental Tables S1-S5
- Suppl.Fig.S1-S7 - Supplemental Figures S1-S7
