# Supplementary material for: Streptococcus pyogenes Infection and the Human Proteome with a Special Focus on the Immunoglobulin G-cleaving Enzyme IdeS
Source: Mol Cell Proteomics. 2018 Mar 6;17(6):1097–111. doi: 10.1074/mcp.RA117.000525 (PMC5986240; doi:10.1074/mcp.RA117.000525)
Supplement: Supplemental Data [file supp_RA117.000525_134376_1_supp_77688_p48xws.pdf]

Supplementary Figures S1-S7

Streptococcus pyogenes infection and the human proteome with a special focus on the IgG-cleaving enzyme IdeS

Christofer Karlsson[1], Sofia Järnum[2], Lena Winstedt[2], Christian Kjellman[2], Lars Björck[1], Adam Linder[1] and Johan Malmström[1]

1. Lund University, Division of Infection Medicine, Department of Clinical Sciences, Lund, Sweden
2. Hansa Medical AB, Lund, Sweden

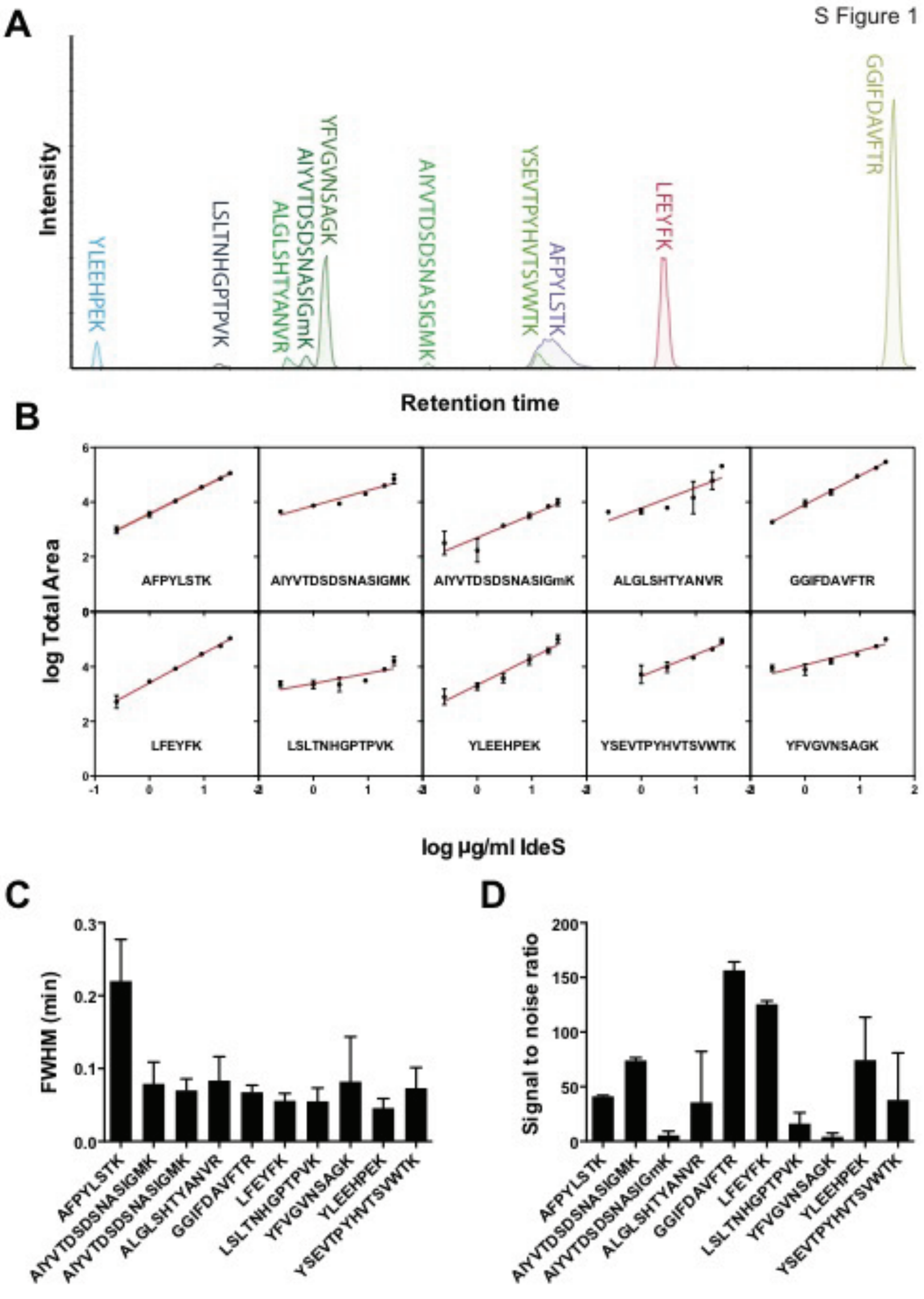

Figure S1. Development of SRM assays targeting streptococcal IdeS. Recombinant IdeS was serially diluted into two human serum backgrounds and then trypsin digested follow ed by SRM-MS analysis using IdeS assays (n=10) from Karlsson et al. 2012.

A) Total transition area (intensity) for indicated peptides over retention time at the highest IdeS concentration.

B) Response curves for all peptides ± SD. The red line shows regression.

C) Average peptide peak full width at half maximum (FWHM) ± SD.

D) The peptide signal to noise ration calculated from integrated peak area divided by background signal.

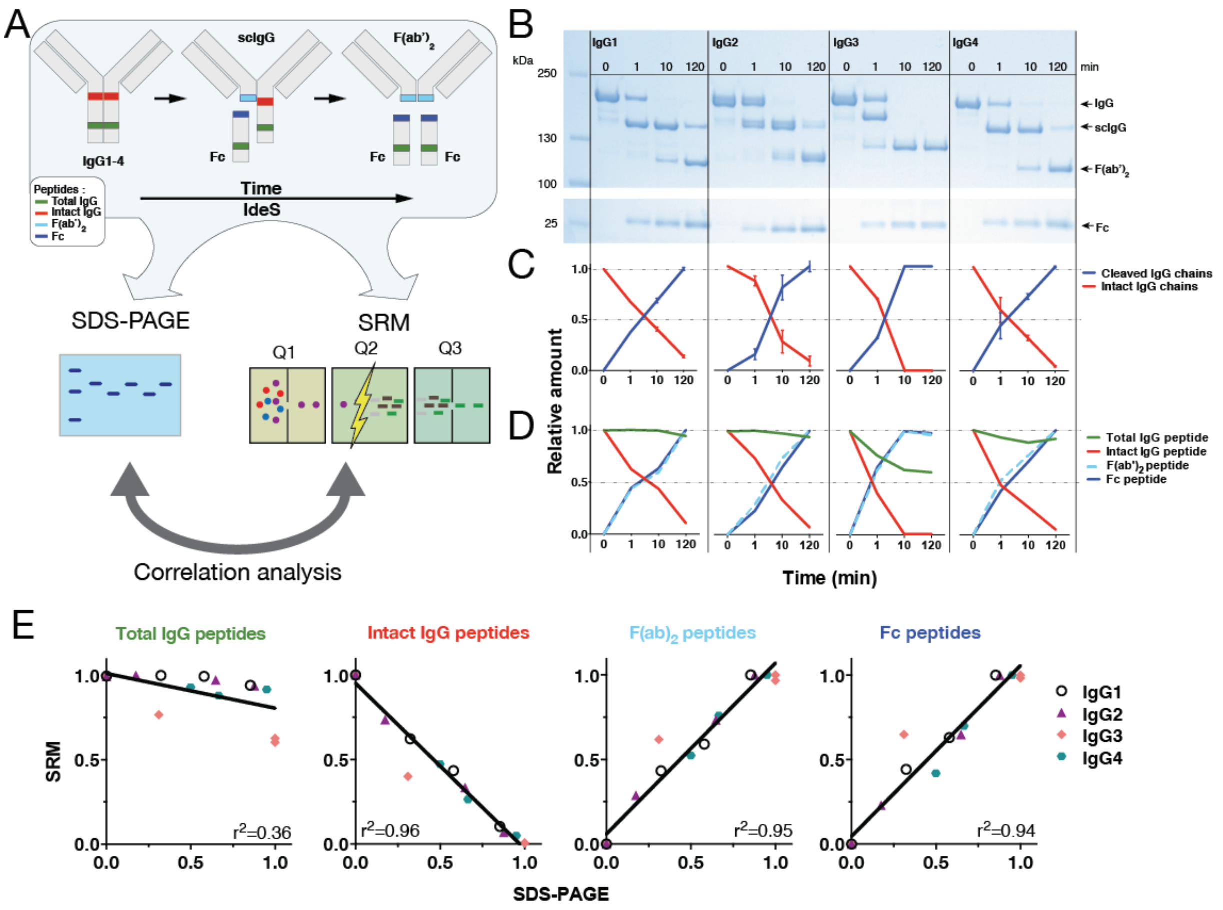

Figure S2. Time course IdeS cleavage of monoclonal IgG *in vitro*.
A) IdeS cleavage of IgG is a two-step sequential process first forming first singly cleaved IgG (scIgG) and one Fc fragment followed by full digestion into a F(ab')$_2$ fragment and two Fc fragment. With this experiment, we correlated IgG integrity from subclasses 1 to 4 (quantified by SDS-PAGE gel-band densitometry measurements) with SRM measurements of tryptic peptides targeting total IgG, IdeS cleaved IgG or intact IgG (see Table S2).
B) Non-reducing SDS page showing IdeS cleavage of IgG subclasses over time. Gel-band annotations are on the right side and molecular weight standards (kDa) on are the right side. IgG subclass and Time points are indicated above. c) Show the estimated values of IgG integrity calculated from densitometry measurements of gel-band intensities. Data shown is averaged ± SD from two different models (not shown). d) SRM analysis of tryptic digests of same samples as in b). Peak total areas are all normalized to the maximum intensity of each subclass and peptide respectively. Peptide sequences are found in Table S2. e) Correlation (r^2) of SDS-PAGE c) and SRM results d).

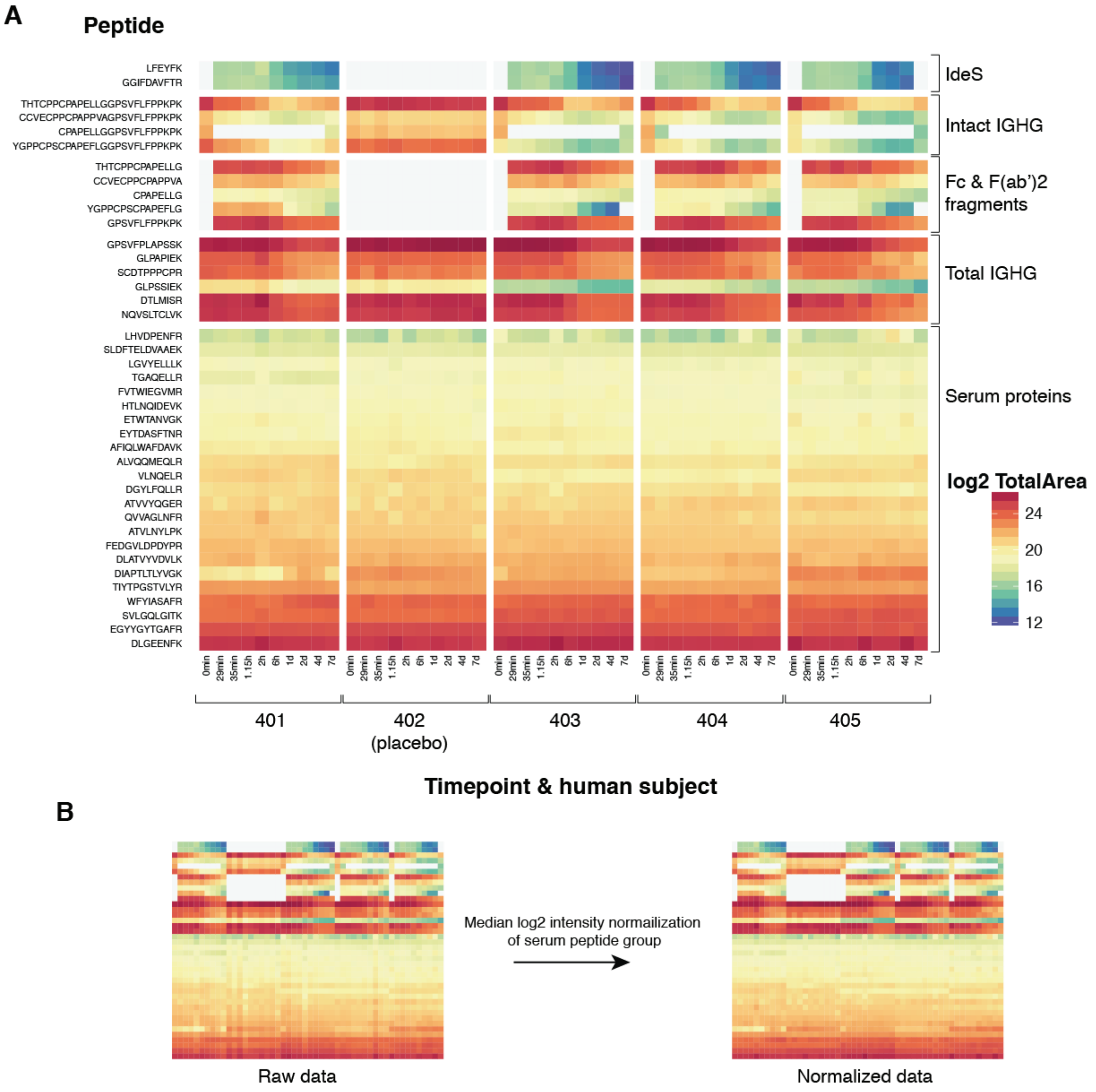

Figure S3. SRM analysis of time course human serum samples from the clinical Phase I trial of IdeS. Four human subjects (401, 403-405) were treated with 0.12 mg IdeS per kg bodyweight or placebo treated (subject 402). Serum samples were obtained before IdeS administration (0 min) and at 9 time points after IdeS administration.

A) These 50 samples were trypsin digested and analyzed with SRM-MS using assays for the indicated peptides and peptide/protein groups. Log2 peptide transition total area is plotted with colored tiles. The data is normalized based on median log2 intensity values
of serum peptide group.

B) Shows raw (Table S3) and normalized data (a)

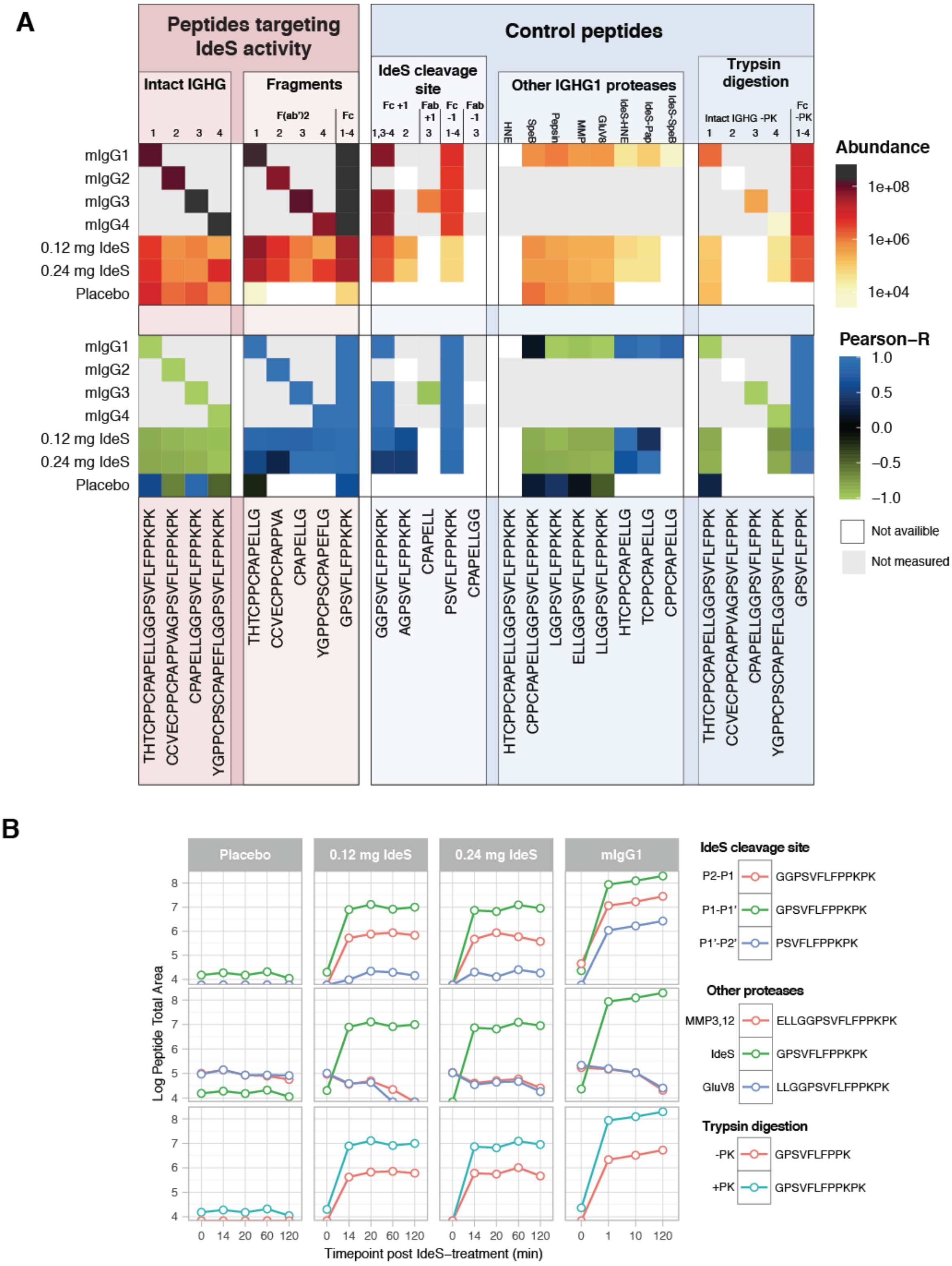

Figure S4. SRM analysis of peptides targeting IdeS activity and control peptides *in vitro* and *in vivo*. The *in vivo* samples are from the IdeS clinical trial first 5 five time points (where the total IgG levels are similar, see Figures 2B-D) and *in vitro* samples from monoclonal IgG treated with IdeS (see Figure S2B).
A) Two groups of peptides were tested (see Table S2): red right panels are peptides targeting IdeS activity and blue left panel control peptides hypothetically not associated with IdeS activity. The subgroups of control peptides are as follows; **IdeS cleavage site**: Peptides generated by offset IdeS cleavage and onset trypsin cleavage (IdeS sites P2-P1 or P1'-P2' on IgG heavy chains); **Peptides generated by other IGHG1 proteases** and trypsin cleavage; and **Trypsin digestion** peptides without a C-terminal PK residues and low-probability trypsin cleavage residues. The yellow-red-black heatmap data shows the summed peptide

intensities over the time points (abundance) and the green-black-blue show Pearsons correlation coefficient of respective peptide and IgG Fc peptide (GPSVFLPPKPK) across time points for respective subclass i*n vitro* or human subject.
B) Shows examples of data included in A).

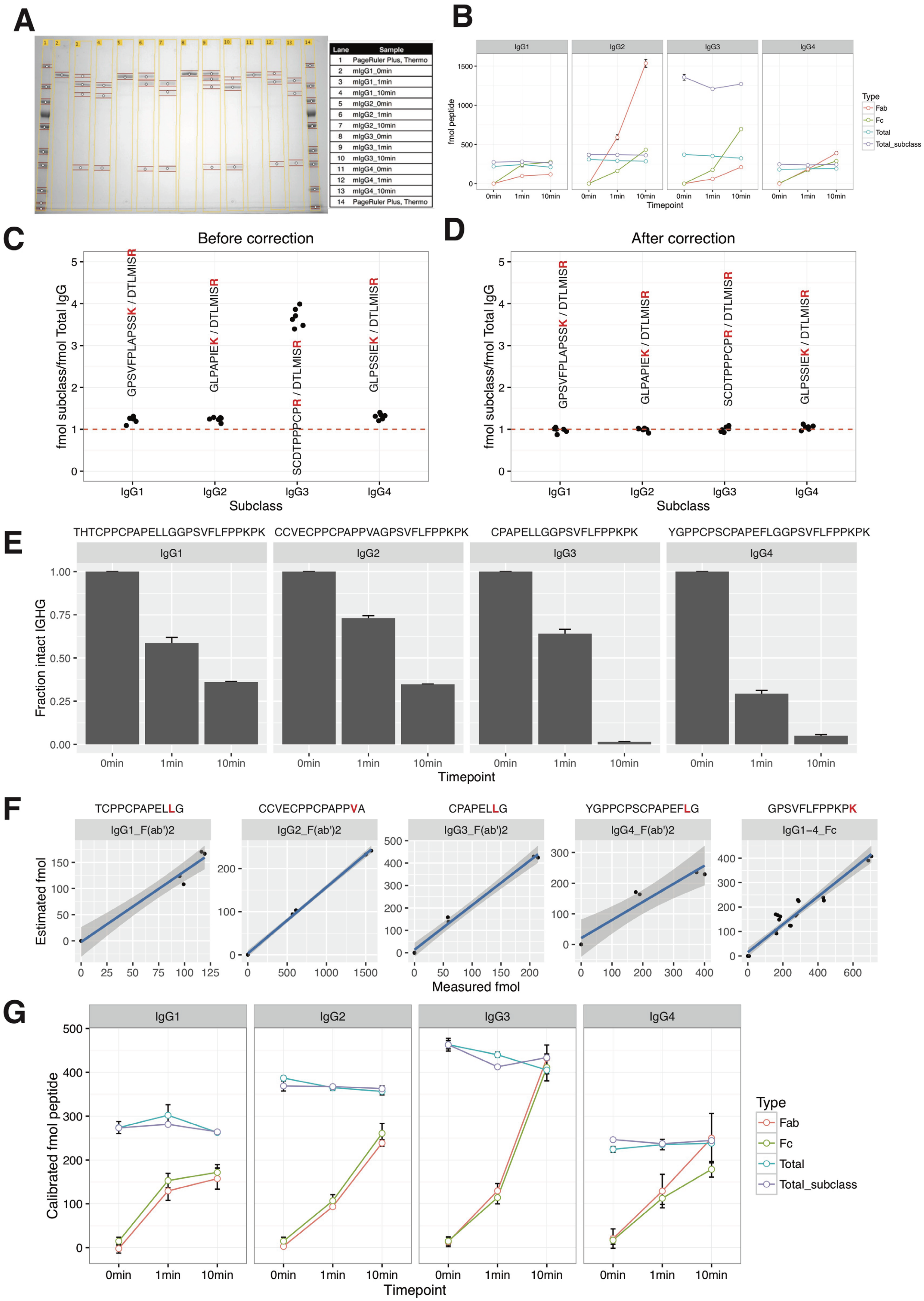
A
Lane Sample
1 PageRuler Plus, Thermo
2 mIgG1_0min
3 mIgG1_1min
4 mIgG1_10min
5 mIgG2_0min
6 mIgG2_1min
7 mIgG2_10min
8 mIgG3_0min
9 mIgG3_1min
10 mIgG3_10min
11 mIgG4_0min
12 mIgG4_1min
13 mIgG4_10min
14 PageRuler Plus, Thermo
B
IgG1
IgG2
IgG3
IgG4
fmol peptide
Timepoint
0min
1min
10min
Type
Fab
Fc
Total
Total_subclass
C
Before correction
fmol subclass/fmol Total IgG
GPSVFPLAPSSK / DTLMISR
GLPAPIEK / DTLMISR
SCDTPPPCPR / DTLMISR
GLPSSIEK / DTLMISR
Subclass
D
After correction
E
THTCPPCPAPELLGGPSVFLFPPKPK
CCVECPPCPAPPVAGPSVFLFPPKPK
CPAPELLGGPSVFLFPPKPK
YGPPCPSCPAPEFLGGPSVFLFPPKPK
Fraction intact IGHG
Timepoint
F
TCPPCPAPELLG
CCVECPPCPAPPVA
CPAPELLG
YGPPCPSCPAPEFLG
GPSVFLFPPKPK
IgG1_F(ab')2
IgG2_F(ab')2
IgG3_F(ab')2
IgG4_F(ab')2
IgG1−4_Fc
Estimated fmol
Measured fmol
G
Calibrated fmol peptide
Timepoint
Type
Fab
Fc
Total
Total_subclass

Figure S5. Heavy peptide calibration using IdeS-treated purified monoclonal IgG1-4. Monoclonal IgG1-4 was treated with IdeS, for 0, 1 or 10 min.

A) SDS-PAGE analysis of samples.

B) The samples were also digested with trypsin and spiked with heavy peptides (see Table S2). The figure shows the uncorrected absolute light peptide amounts over time.

C) and D) Corrections of total IgG subclass concentrations against peptide DTLMISR concentrations.

E) Fractions of intact IgG subclass over time points calculated from peptides intensities show above panels.

F) Linear regression models of estimated fmol $F(ab')_2$ peptides (Panel 1-4) or Fc peptide (Panel 5) calculated from values in E) multiplied with total IgG amounts in C) against the measured amounts of peptides indicated above each panel.

G) Shows the light peptide amounts using the obtained corrected of heavy peptide amounts. All samples were digested in two replicate samples. B) E) and G) shows average values ±SD. Red peptide residues in peptides sequences are the heavy labelled position in reference peptide.

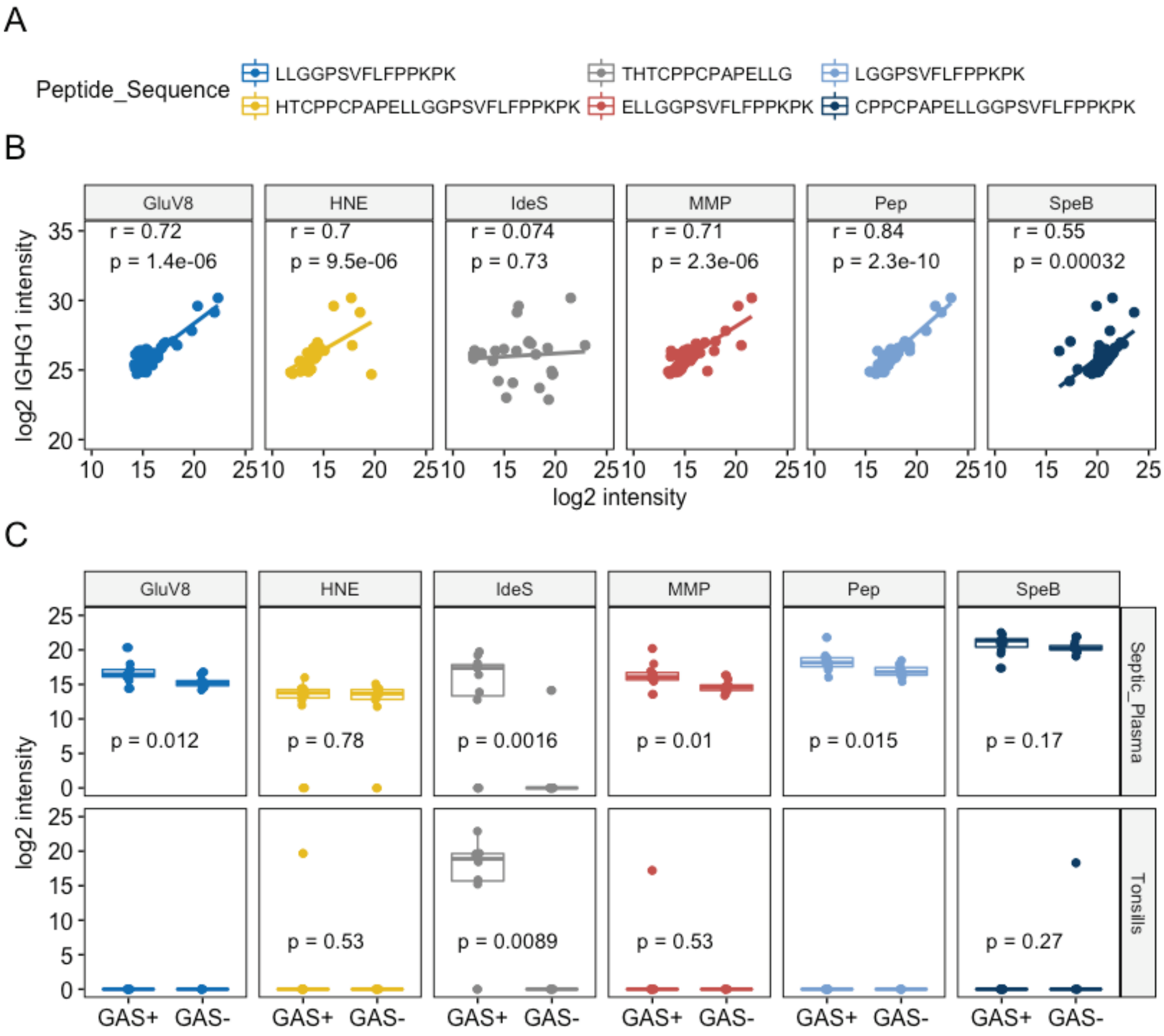

Figure S6. Analysis of peptides targeting proteolytic activity on IgG1 heavy chain in human samples. Tryptic digests of proteins from human infection samples and control samples (see Figures 3A and 5) were analyzed with SRM and the data shown is label-free quantified.

A) The shown peptides are hypothetically generated by selective IgG1 heavy chain protease activity and trypsin cleavage. The proteases, target peptides and abbreviations are as follows: Staphylococcal Glutamyl endopeptidase GluV8 (LLGGPSVFLFPPKPK, GluV8), Human Neutrophil elastase (HTCPPCPAPELLGGPSVFLFPPKPK, HNE), Streptococcal IdeS (THTCPPCPAPELLG, IdeS), Human Matrix Metalloproteinase-3 (ELLGGPSVFLFPPKPK, MMP), Human Pepsin (LGGPSVFLFPPKPK, Pep), Streptococcal SpeB (CPPCPAPELLGGPSVFLFPPKPK, SpeB) and are indicated with respective colors and abbreviations in below panels.

B) Scatter plot of peptide intensities in a) (x-axis) and IgG1 heavy chain (IGHG1) total levels (peptide GPSVFPLAPSSK, y-axis) in all human samples (see Figure 5). Missing values are not shown. Correlation coefficient, r, and p-values, p, are indicated in panels and are determined with Spearman's method.

C) Samples from the following selected patient groups are shown: septic plasma and bacteremia with *S. pyogenes* (GAS+) or other species (GAS-) in upper panel; tonsillar swabs from patients with *S. pyogenes* tonsillitis (GAS+) or healthy controls (GAS-) in lower panel. The peptide means intensities of GAS+ or GAS- were compare2.321928095d with Wilcox's test and the corrected p-values are indicated. Missing values are replaced with 0.

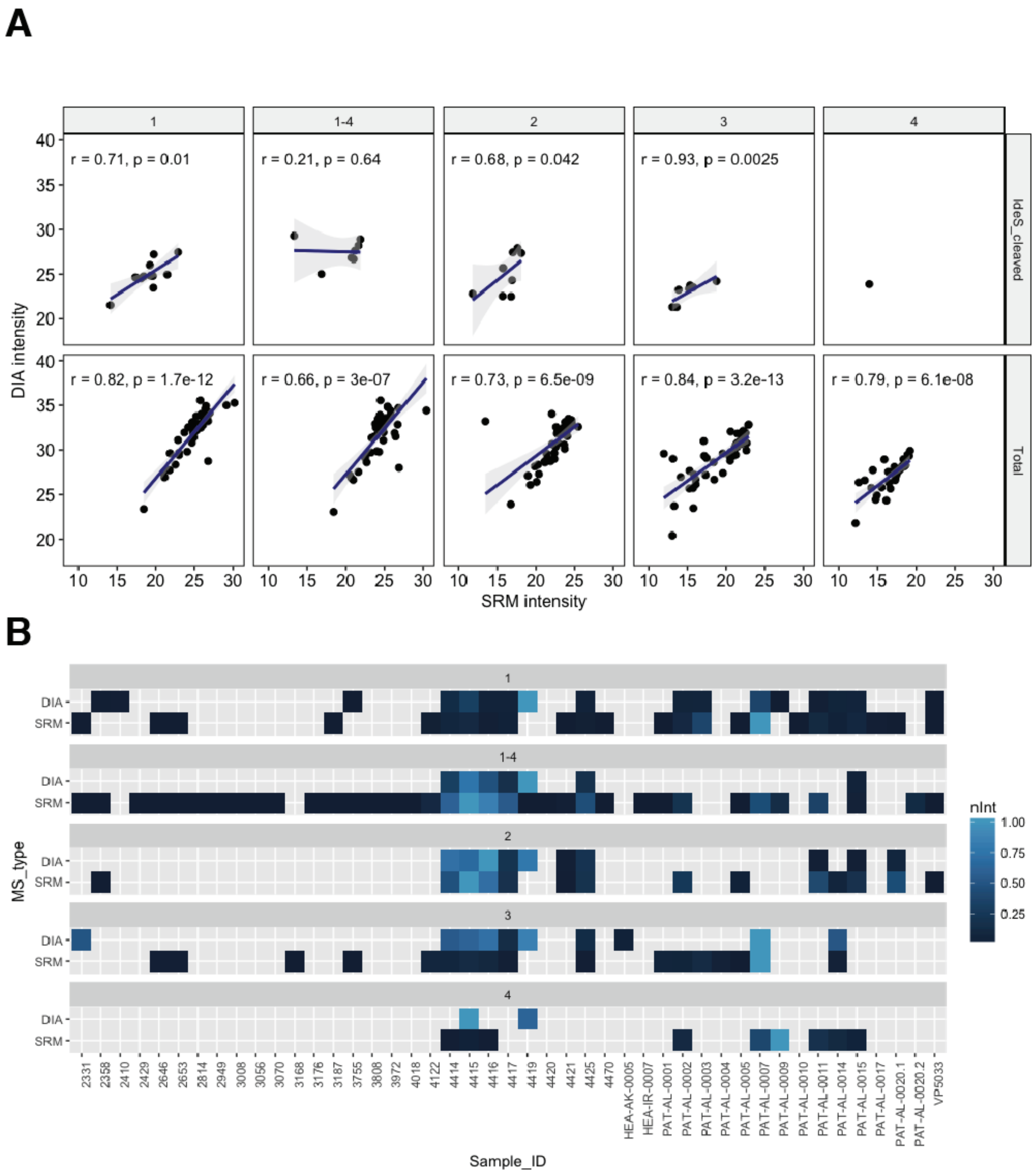

Figure S7. Comparison of SRM and DIA intensities for peptides targeting IgG levels and IdeS proteolytical activity. DIA data files from patient samples (Table S1) were queried for peptides targeting the semi-tryptic IgG peptides: THTCPPCPAPELLG, CCVECPPCPAPPVA, CPAPELLG, YGPPCPSCPAPEFLG and GPSVFLFPPKPK (proxy for IdeS activity on IgG1-4) and also tryptic peptides located distally from the IgG hinge region: DTLMISR, GPSVFPLAPSSK, GLPAPIEK, SCDTPPPCPR, and GLPSSIEK (proxy for total IgG1-4 levels) using the developed SRM assays (see Table S2). The DIA chromatograms for each peptide and sample were manually analysed with Skyline and considering the RT and similarity to the library spectrum (dotp score) as the major discriminants for peak integration or peak rejection. The resulting DIA data was compared to existent SRM data (Figure 5 and Table S5) and peptide intensity was defined as integrated light peptide fragment areas for both SRM and DIA. A) Scatter plots showing the spearman correlation (r) and p values (p) of individual peptides between SRM (x-axis) and DIA (y-axis) intensities faceted by peptide group and IgG subsclass selectivity. B) Shows the normalized intensity per semi-tryptic IgG peptide targeting IdeS activity per sample (x-axis, see Table 1 for sample details)) and MS type (y-axis) faceted IgG subsclass selectivity.
